# Effect of ceramic reinforcements in TIG-welded Al/SiCp and Al/TiB_2_ composites for enhanced mechanical properties

**DOI:** 10.1038/s41598-026-35715-y

**Published:** 2026-01-16

**Authors:** R. Ganapathy Srinivasan, M. Bakkiyaraj, C. Rajaravi, M. Selvam, Robert Cep, B. Swarna

**Affiliations:** 1 Department of Mechanical Engineering, Vel Tech Multi Tech Dr Rangarajan Dr Sakunthala Engineering College, Chennai, 600062 India; 2 Department of Mechanical Engineering, Rajalakshmi Institute of Technology, Chennai, 600124 India; 3 Department of Mechanical Engineering, Hindhusthan College of Engineering and Technology, Coimbatore, 641032 India; 4 Department of Machining, Assembly and Engineering Metrology, Faculty of Mechanical Engineering, VSB-Technical University of Ostrava, 70800 Ostrava, Czech Republic; 5 Department of Biosciences, Saveetha School of Engineering, Saveetha Institute of Medical and Technical Sciences, Chennai, 602105 India

**Keywords:** TIG welding, Al/SiCp, Al/TiB₂, Microstructure analysis, Mechanical properties, Engineering, Materials science

## Abstract

This research determines the effect of various ceramic particles (SiCp and TiB₂) on the Tungsten Inert Gas (TIG) welding of Al/TiB₂ and Al/SiCp composites. The reinforcement concentrations of 2%, 4% and 6% were chosen for the investigation for both SiCp and TiB₂ particles, and their effect on tensile strength (TS), hardness, and microstructure was studied using appropriate instruments. The SEM microstructure confirmed the ceramic particles distribution of different composite materials. The microstructure of the welds varies depending on the composition of the Al/TiB₂ and Al/SiCp composites. XRD analysis displayed that the phase compound formed in weld metal is similar to the metal matrix composites (MMCs), indicating strong bonding between the reinforcements and the matrix. XRD analysis confirmed the retention of reinforcement phases without the formation of undesirable reaction products, indicating good metallurgical compatibility and stable interfacial bonding between the matrix and reinforcements. Addition of TiB₂ and SiCp ceramic reinforcements into the aluminum matrix significantly improved the hardness of the welded joints. The highest hardness value of 172.8 HV was obtained for the Al/SiCp composite containing 4 wt% SiCp, representing an improvement of 25.94% and 8.88% compared to the composites containing 2 wt% and 6 wt% SiCp, respectively. Which is greater than 25.94% and 8.88% compare to 2% and 6% respectively. Similarly, the Al/TiB₂ MMC containing 4% TiB₂ showed the maximum hardness value of 98.3 Hv, followed by the composites containing 2% (85.9 HV) and 6% (74.6 HV) TiB₂, respectively. Which is greater than 14.44% and 24.11% compare to 2% and 6% respectively. This result shown that the inclusion of reinforcement particles increases the hardness of the weld zone, with the optimum reinforcement being 4% of SiCp and 6% of TiB₂. The welded specimens were tested using tensile tester to determine their TS and ductility. The results revealed that the inclusion of ceramic particles increases the TS of the welds but ductility found to be deceased. The Al/SiCp MMCs with 4% displayed the highest TS of 226.7 MPa followed by those with 2% and 6% respectively. Which is increase 8.16% and 3.37% compare to 2% and 6% respectively. Similarly, the aluminium based MMCs containing 4% TiB₂ recorded the highest TS of 228.5 MPa followed by the Al/TiB₂ MMCs containing 2% and 6% TiB₂ respectively. Which is increase 11.35% and 4.77% compare to 2% and 6% respectively. However, the Al/SiCp MMCs with 4% SiCp showed relatively lower ductility of 2.9% when compared to the Al/TiB₂ MMCs with 4% TiB₂. Overall, the inclusion of ceramic reinforcements into the aluminium based MMCs improves the mechanical performance of the weldments, although the optimal composition depends on specific application requirements.

## Introduction

MMCs have gotten significant attention from scientists in recent years and are recognized as advanced materials because of their excellent mechanical and functional properties. Their growing importance comes from their potential to serve as alternatives to conventional metals and alloys enabling diverse applications sector such as automotive, aerospace and electronics. The production of MMCs typically involves incorporating ceramic or metal particles or fibers into a metal matrix to improve the specific mechanical and physical properties. Among the different MMCs, the aluminium-based MMCs have materialized as prominent system because of their lightweight, exceptional corrosion resistance, and high strength-to-weight ratio^1^. TIG welding has proven to be an exceptionally versatile technique for joining MMCs. Nevertheless, the reinforcements like ceramic particles or fibers introduces an intricate challenge in welding that greatly influence the weldability, and mechanical performance of the weldments^2^.

Cheng et al.^3^ reported that the fusion-welded TiB₂ ceramic particle-reinforced aluminium MMCs joints significantly improves their mechanical properties. The formation of Al₃Ti third-phase compound at the weld interface led to the improved wettability, as reported by the authors. In addition, the unweldable composite was also examined in order to understand the ductile behaviour and its results showed that the MMCs containing an optimum concentration of TiB₂ reinforcements exhibited superior ductile fracture features and improved mechanical strength when compared to those without ceramic reinforcements^4^. Bo et al.^5^, studied hoe using AA4043 filler wire in TIG welding affects the AA6082 alloy, focusing on the combined impact of strontium (Sr), and titanium (Ti) inclusions on mechanical properties. They found that adding both Ti and Sr improved the fatigue and TS of the welded samples and helped reduce the cracking and porosity. Adding a small amount of magnesium (Mg) to the filler wire also led to better tensile properties in the TIG-welded Al-Mg2Si alloy joints and supported stronger precipitation hardening. Detailed research on the mechanical and metallurgical properties of these welded joints was reported in^6–8^. Fattahi et al.^9^, used ultrasonic vibration during TIG welding process to fabricate aluminum joints reinforced by nano particle dispersion. They found that this method reduced porosity and improved the TS of the joints at a significant level. Another study examined how adding rice husk to aluminium-based MMCs affected weldability using TIG welding. The results showed that machinability, hardness, and wear resistance improved when rice husk was included in the aluminium matrix. Dinaharan et al.^10^, also investigated the wear behaviour and metallurgical features of friction stir processed copper composites with rice husk ash. They found that adding rice husk particles improved wear resistance and mechanical properties, with FSP ensuring uniform particle distribution in the aluminium matrix. A hybrid nanocomposite of Al/ZrC/TiC joints produced by TIG welding though accumulative roll bonding, showed enhanced mechanical properties without cracking or porosity^11^. Another study on an A356 + 15% SiCp cast composite manufactured by friction stir welding (FSW) caused partial dissolution of SiC particles, the joint exhibited strong tensile properties^12^.

A recent study^13^, examined the mechanical characteristics of Al3003 alloy welded joints reinforced with titanium dioxide [TiO_2_], nanoparticles using gas tungsten arc welding (GTAW). The addition of TiO_2_ nanoparticles significantly increased the microhardness, TS and ductility of the welded specimens. Wang et al.^14^, investigated composite coatings produced by in-situ formation of titanium carbide (TiC) reinforcements within a ferrite-based matrix. The researcher applied a multi-pass overlapping GTAW deposition technique to create the coating on the substrate. The resulting layers exhibited enhanced hardness, fatigue and wear resistance. The presence of titanium and boron was identified as the primary factor contributing to microstructure refinement. Fattahi et al.^15^ studied the reinforcement of aluminum TIG welding metals using a CNT/aluminium composite wire as filler. They found that adding CNTs particles to the aluminium matrix improved the weld joints mechanical properties and led to a more even distribution of reinforcement. Varshney and Kumar^16^, reviewed different grades of aluminum alloy, focusing on their strength, workability and the optimization of welding parameters. They concluded that appropriate selection of process parameters, filler materials and heat treatment methods can greatly enhance the weldability and performance of MMCs.

A similar study examined the coating effects on the mechanical behaviour of TIG welded black steel pipes using carbon fibre powder reinforced composites^17^. The results showed substantial enhancement in both TS and corrosion resistance of the welds. In contrast, limited research on the laser-based TIG welding techniques^18–21^ demonstrated that this process produces unform defect free joints with superior mechanical properties. Gupta et al.^22^ evaluated the mechanical properties of TIG-cladded in situ TiC-TiB₂ powder reinforced composite coating on AISI 304 stainless steel, finding that cladding enhanced mechanical properties and achieved even particles distribution within the matrix. Another study on TIG surface alloying to fabricate Al/SiC 304 stainless steel reported increased hardness, wear resistance, and corrosion resistance^23^. The improvement of mechanical properties in composites by adding ceramic particles or fibres in well documented. However, the effects of these additions on weldability and performance during welding are not fully understood. TIG welding is a versatile method for joining MMCs, but there is limited information on the optimal composition for effective TIG welding that also maximize mechanical properties. This investigation aims to discourse the gap by the effects of KBF_4_, K_2_TiF_6_, and SiCp addition to aluminum-based MMCs reinforced with SiCp and TiB₂ particles.

## Experimental method

This research investigation employed A356 aluminum alloy as the base metal to manufacture the aluminium-based MMCs with two unique reinforced combinations of silicon carbide (SiCp) and titanium diboride (TiB₂) particles. The synthesis uses two salts, namely, potassium tetrafluoro borate (KBF_4_) and potassium hexafluoro titanate (K_2_TiF_6_). The A356 alloy liquid metal was held at 750 °C for 30 min; subsequently, 15 g of magnesium was added to the molten alloy. The melt was stirred for 30 min under argon protection to allow complete in-situ formation of TiB₂ from KBF₄ and K₂TiF₆ and to ensure uniform particle distribution. The stirring was carried out at a speed of 450 rpm using a mechanical impeller^12^. Finally, the molten metal was then cast into a sand mold cavity to form the SiCp-reinforced MMCs. The same procedure was followed to fabricate Al/SiCp composites with different proportions of SiCp, containing 2%, 4%, and 6% respectively, allowing for an investigation in order to examine how the SiCp concentrations influences the performance of the Al/SiCp composites. Similarly, TiB₂ reinforced Al/ TiB₂ composites with 2%, 4%, and 6% concentrations were fabricated. TiB₂ particles were synthesized in situ through chemical reactions between KBF₄ and K₂TiF₆ salts in molten aluminum. SiCp particles were externally added (ex situ) to the molten A356 aluminum alloy in predetermined weight percentages (2%, 4%, and 6%) under mechanical stirring.

**Fig. 1:**
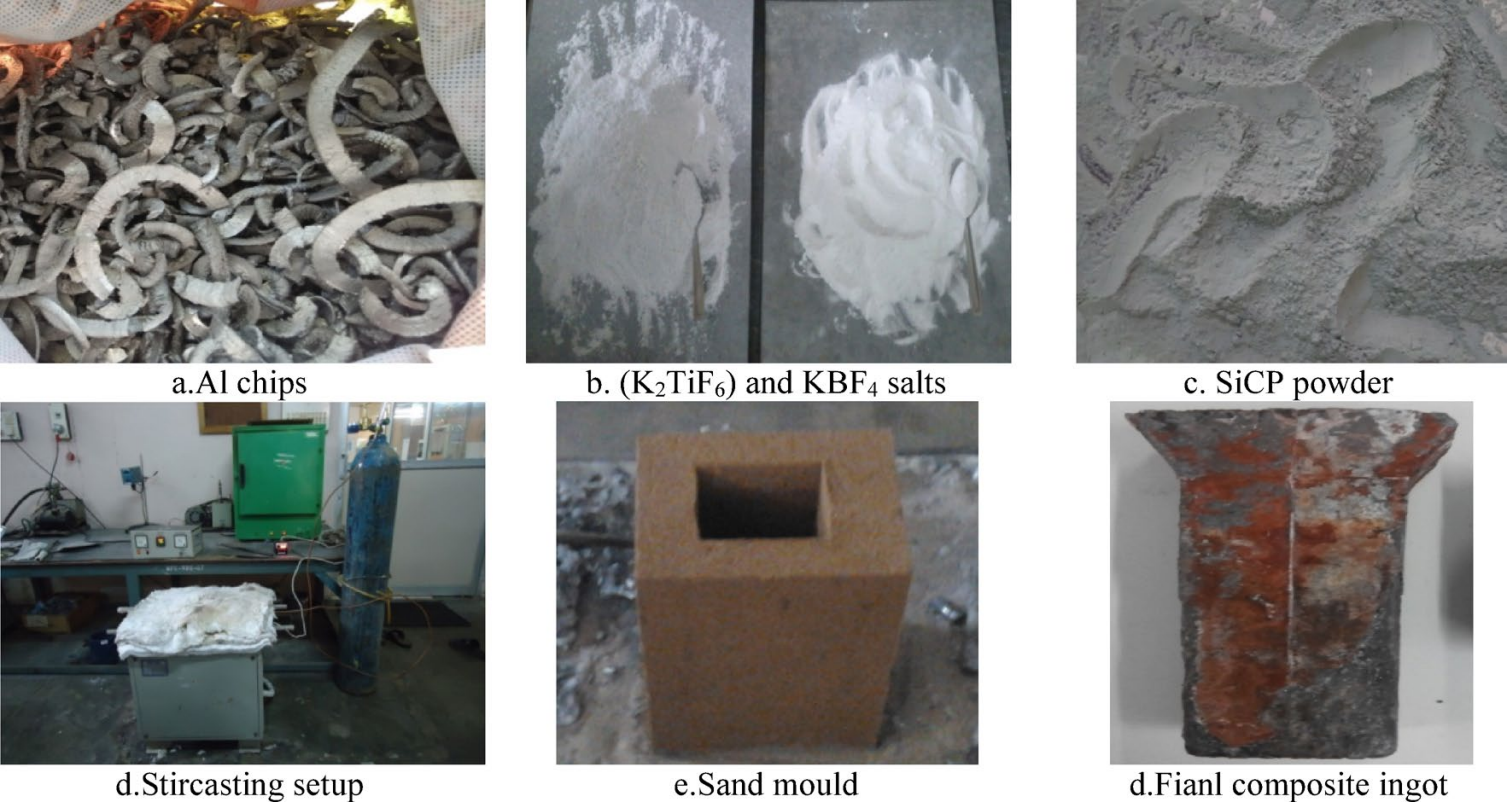
Initiating materials and final product of composite.

In the presence of molten aluminium, these salts react, leading to the synthesis of titanium diboride (TiB₂) particles in the melt. The process includes the removal of slag before pouring the mixture into the mold. The resulting Al/TiB₂ melt is cast using both sand mold, and the castings are produced at various pouring temperatures, ranging from 820 °C. To create aluminium matrix composites (Al/TiB₂ MMCs), the A356 aluminium alloy is combined with 2%, 4%, and 6% of KBF_4_ and K_2_TiF_6_ by weight%. Stirring is employed to ensure homogenous dispersal of the ceramic’s reinforcements in the entire matrix metal. The use of a sand mold for casting implies a focus on the quality and integrity of the final MMC as shown in the Fig. 1a–c. The powders are introduced into the molten aluminium alloy at 700 °C under continuous stirring with a mechanical stirrer, as illustrated in Fig. 1d–c. Subsequently, the molten mixture is solidified into a sand mold cavity to form MMCs with size of 100 mm × 100 mm × 2 mm.

**Fig. 2:**
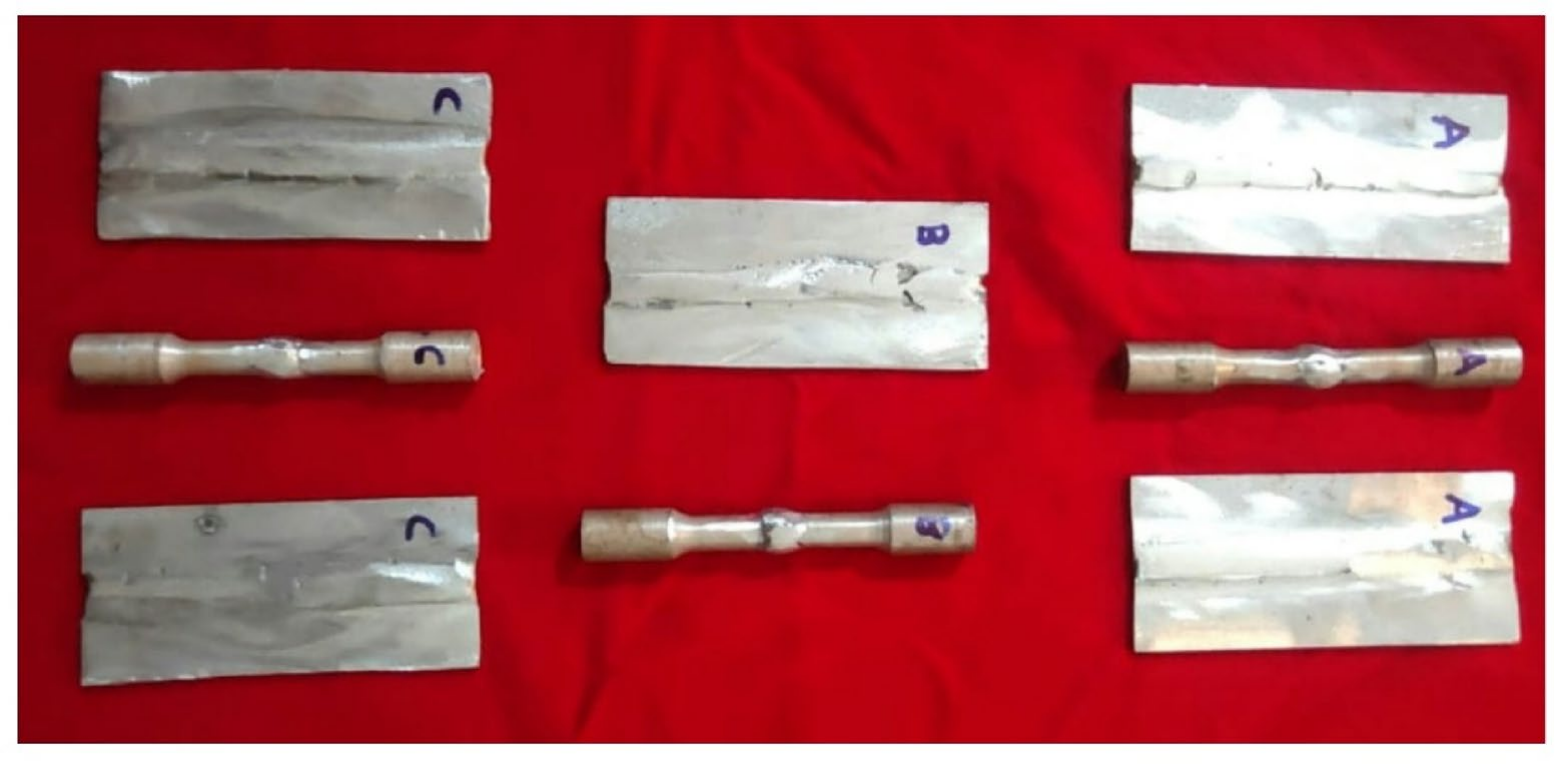
Welded samples of Al/TiB₂ MMC and Al/SiCp MMCs.

TIG welding was performed using 180 A, 20 V and 1000 mm/min because these parameters provided stable arc characteristics, sufficient penetration for A356 MMCs, and minimized heat-affected zone grain coarsening [27 & 31]. In the TIG welding, a tungsten electrode is used to generate heat by creating an arc between the work piece and the electrode. The work piece is placed in an argon atmosphere to avoid oxidation. The prepared MMCs were subjected to microstructural analyse using scanning electron microscopy (SEM). The phase composition of the welded specimens was investigated using an X-ray diffractometer (XRD) model of Rigaku Ultima IV. The Vickers hardness test was employed to calculate the hardness value of the composite with a load range of 1 kg for 15 s and similarly the Universal Testing Machine (UTM) was utilized to determine the tensile behaviour of the welded composites, in which the ASTM standard E8-16 guidelines were followed^24,25^. A crosshead speed of 5 mm/min was maintained during the experimentation. Three samples are tested for each composition, and the average values of the test results were used to determine the mechanical properties (Fig. 2).

## Results and discussion

### Microstructure study of Al/TiB₂ MMCs and Al/SiCp MMCS

**Fig. 3:**
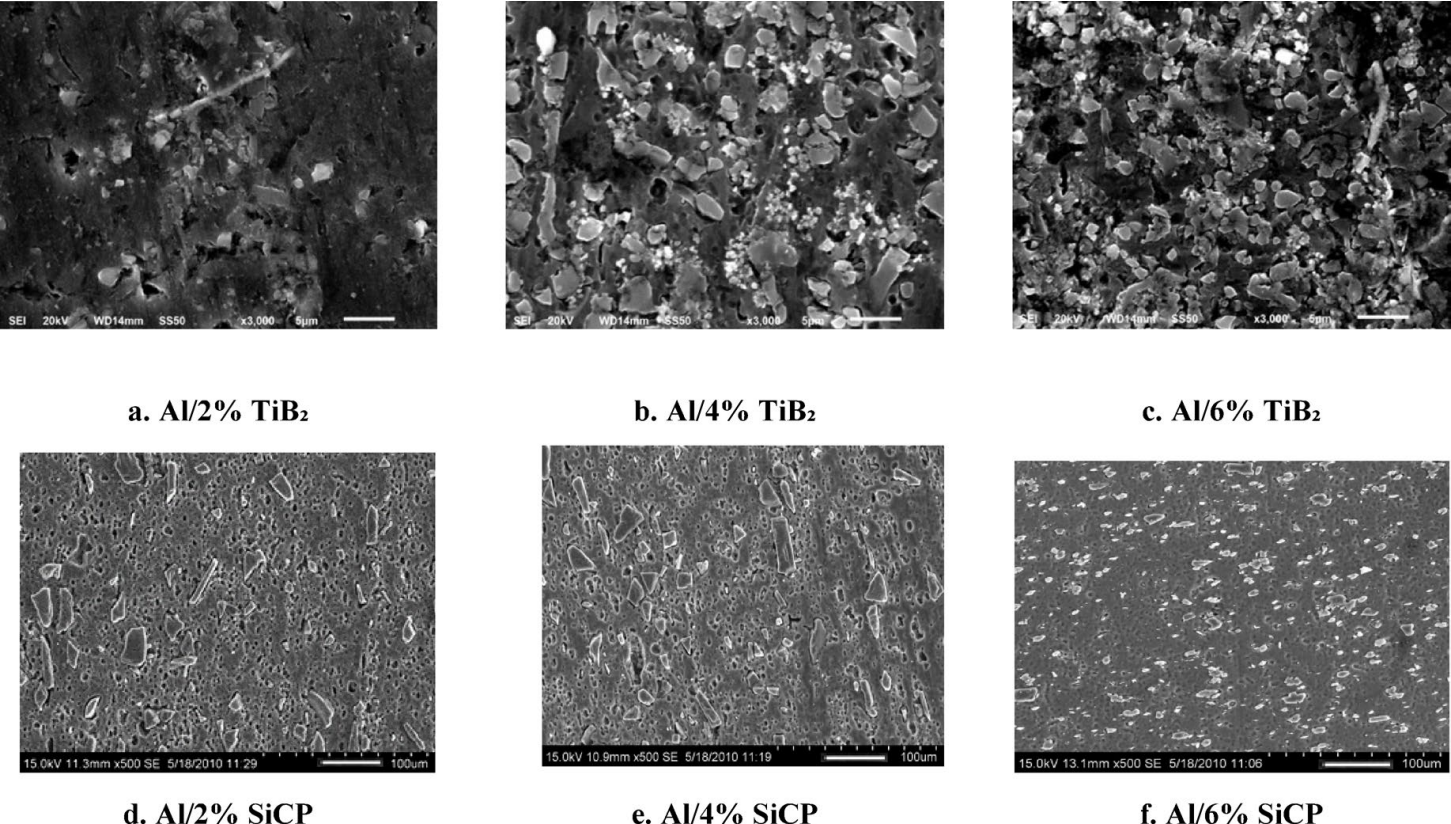
Microstructure analysis Al/TiB₂ MMC and Al/SiCp MMCS.

The Fig.3a–f shows the Al/TiB₂ and Al/SiCp MMC cast in sand mould at 2% 4% and 6% of particles. Microstructure of TIG-welded Aluminum MMCs reinforced with SiCp and TiB₂ particles indicates obvious dependence on the concentration of particulates. Both composite systems have a fairly non-uniform particle distribution in weld zone at 2 wt%. The microstructure of Al/2% TiB₂ has areas of sparse micro-dispersion of reinforcement, which leads to heterogeneous grain refinement and isolated clusters of un-melted particles. In the same way, Al/2% SiCp displays partial agglomeration and irregular interfacial transition which means that reinforcement is not adequate to stabilize the melt pool and encourage uniform solidification. Both Al/TiB₂ and Al/SiCp composites exhibit the best and uniform microstructural morphology at 4 wt% reinforcement. The Al/4% TiB₂ weld zone has fine, equiaxed grains and the TiB₂ particulates are well dispersed, implying that the nucleation efficiency is better. The high particle density causes an effectual limitation to the growth of the grain and improves the uniformity of solidification. The microstructure in Al/4% SiCp displays uniform dispersion of SiC particulates with a low level of clustering which forms the refined dendritic structures and stable interface. This concentration seems to represent the optimal compromise between sufficient particle concentration to allow refinement of the grain and the minimization of over agglomeration.

The reinforcements particle overfilling at 6 wt%. Al/6% TiB₂ indicates a high degree of particle clustering and localized porosity, which is explained by low wettability and higher viscosity of the melt in a high reinforcement content. These aspects interfere with the fluidity in the welding process and give non-uniform grain structure. Likewise, Al/6% SiCp exhibits agglomerates that are thick and impede the flow and solidification of heat resulting in coarser grain patches and microstructure inconsistency. Stress concentrators are also caused by excess reinforcement, and it may weaken the integrity of welds. Comprehensively, the comparative discussion of all the microstructures shows that the reinforcement of 4 wt% is the most favourable weld-zone property of both SiCp and TiB₂ reinforced aluminum composite. This is the optimum concentration that is required to achieve successful grain refinement, homogenous particle distribution, and stable melt dynamics and prevents the defects of under- or over-reinforcement. In turn, 4 wt% SiCp and 4 wt% TiB₂ may be suggested as the best particulate loading to use in order to obtain the best metallurgical quality in TIG-welded aluminum matrix composites, thus, being the most appropriate compositions to high-performance structural use.

### XRD analysis of Al/TiB₂ MMC and Al/SiCp MMCS

**Fig. 4:**
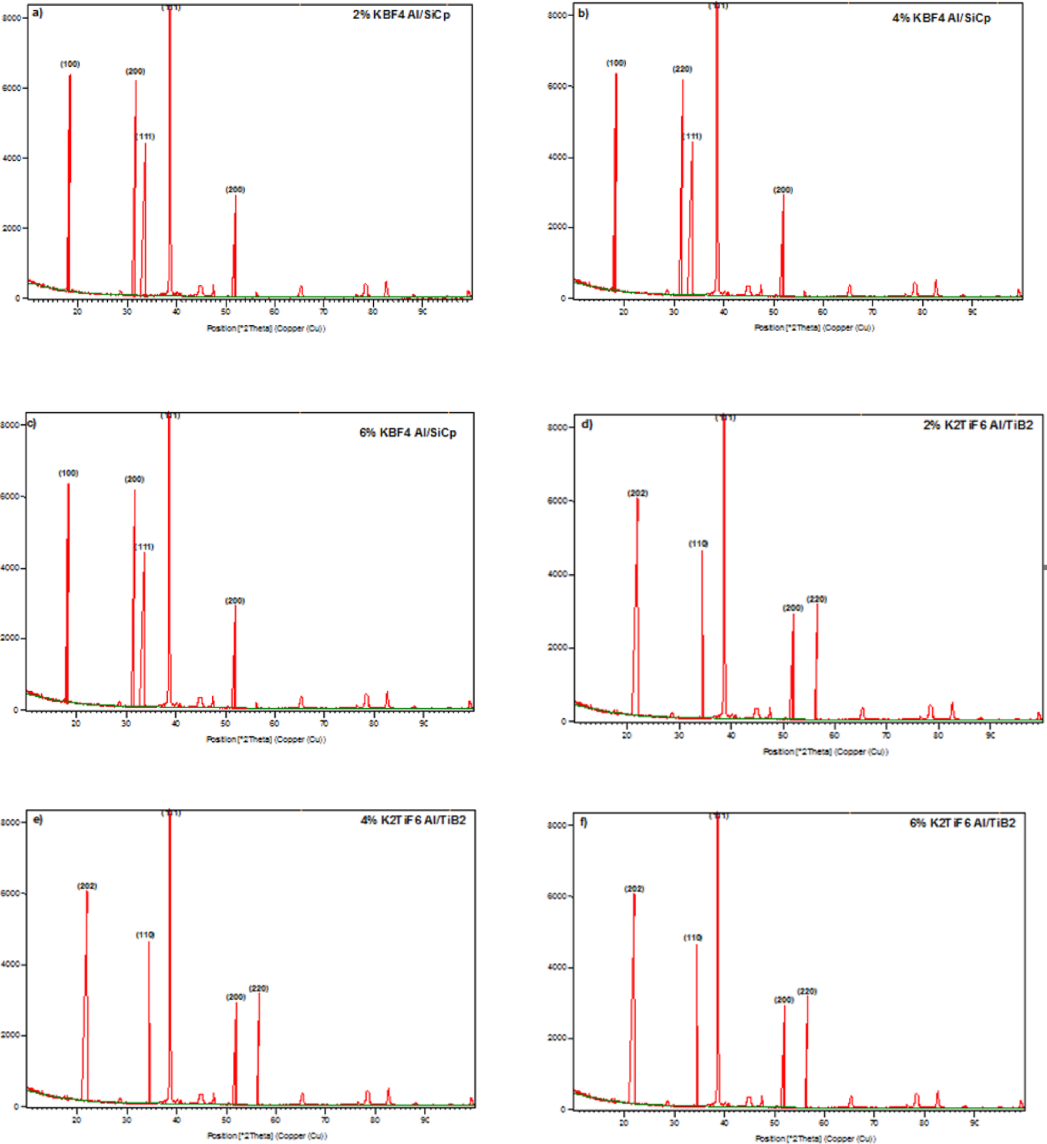
XRD Analysis of welded MMCs with various ceramic concentration **a** 2% of SiCp; **b** 4% of SiCp; **c** 6% of SiCp; **d** 2% of TiB₂; **e** 4% of TiB₂; **f** 6% of TiB₂.

The phases present in the TIG welded metal matrix composites were confirmed with help of XRD analysis as shown in Fig. 4a–1f. The different peaks in the patterns show the various phases present in the composites. The scanning range was from 10° to 90° in 2θ using an interval step of 0.02°. The XRD pattern of the 2% SiCp of Al/SiCp composite (Fig. 4a) exhibited the presence of Al, and SiC, phases. The peaks detected at 2θ = 38.39° and 50.24° are attributed to the (111) and (200) planes of the aluminum (Al) phase, respectively. Meanwhile, the peak at 2θ = 35.13° corresponds to the (111) plane of the silicon carbide (SiC) phase. Additionally, the peaks at 2θ = 15.18° and 32.4° are linked with the (100) and (200) planes, respectively, of the SiC phase. The XRD pattern of the Al/SiC composite containing 4% SiC particles (Fig. 4b) reveals the presence of both Al and SiC phases. The intensities of the peaks related to the Al and SiC phases are higher compared to the composite with 2% SiC^26^.

Particularly, SiC phases are more pronounced in the XRD pattern; specifically, the peaks at 2θ = 38.44° and 50.28° are related to the (111) and (200) planes that represent a strong Al phase. The peak at 2θ = 35.18° is related to the (111) plane, while the peaks at 2θ = 15.19° and 32.83° are linked with the (100) and (220) planes, respectively; all of them also indicate the SiC phase. These higher peaks (15.19°, 32.83°, and 35.18°) at 2θ emphasize the strong incorporation and retention of SiC particles in the Al matrix that’s led to improving the load-bearing capacity. On the other side, effective reinforcement sustains matrix continuity which is essential for maintaining ductility and toughness, while the absence of Al_4_C_3_ intermetallic ensures better interfacial bonding and hindrance to crack propagation. Likewise, for the composite with 6% SiC particles (Fig. 4c), the XRD pattern was clearly showed both SiC and Al phases. The peaks conforming to the SiC phase are also more intense, with the peaks at 2θ = 38.44° and 50.29° linked to the (111) and (200) planes of the Al phase, respectively. The peak at 2θ = 35.18° is associated with the (111) plane of the SiC phase, and the peaks at 2θ = 15.17° and 32.73° are connected with the (100) and (220) planes, respectively, of the SiC phase.

The peak intensities corresponding to the Al and SiC phases become sharper and more intense when compared to the composite with 4% SiC. Hence, the increase in the SiC phase fraction led to the higher wear resistance, hardness, and TS. A slight decrease in TS was noted which may be linked to the SiC clustering that caused crack propagation during the tensile test. This result was in agreement with the previous authors^26,27^. Figure 4.d represents the XRD pattern for the Al/TiB₂ composite consisting of 2% TiB₂. The pattern clearly showed both aluminium (Al) and titanium diboride (TiB₂) phases, and these demonstrated higher intensity than that of the other phase. The peaks at 2θ = 38.41° and 50.23° match planes of (111) and (200) respectively, for the Al phase, and the peaks at 2θ = 36.63° and 56.71° are for the (110) and (220) planes, respectively, of the TiB₂ phase. In addition, the peak at 2θ = 23.72° also matches the (202) plane of the TiB₂ phase. For the 4% Al/TiB₂ composite (Fig. 4e), the XRD pattern shows the existence of Al, and TiB₂. The peaks for the Al and TiB₂ phases are more intense than those of other phases. Specifically, the peaks at 2θ = 38.44° and 50.24° match the planes of (111) and (200) respectively, for Al phase, while the peaks at 2θ = 36.63° and 56.68° match the planes of (110) and (220), respectively, for the TiB₂ phase. Furthermore, the peak at 2θ = 23.70° matches the (202) plane of the other two phases^28^.

The XRD pattern for the 6% Al/TiB₂ composite (Fig. 4f) shows that Al and TiB₂ phases are present. Similar to the other composition, the peaks for the Al and TiB₂ phases are stronger than those for the other phase. The peaks at 2θ = 38.46° and 50.24° that matches the (111) and (200) planes of the Al phase, while the peaks at 2θ = 36.63° and 56.68° for the (110) and (220) planes of the TiB₂ phase respectively. Furthermore, the peak at 2θ = 23.70° also corresponds to the (202) plane of the TiB₂ phase. The present study examined the microstructure and phase analysis of TIG welded (2%, 4%, 6%) Al/SiCp and (2%, 4%, 6%) Al/TiB₂ metal matrix A356 composites. The XRD study revealed that the Al/SiCp composite contained Al and SiC phases, whereas the Al/TiB₂ composites comprised Al and TiB₂, phases. The peaks that matched the reinforcing phases were more intense as the amount of reinforcing increased in the composites. The TIG welding process performed well to manufacture defect-free MMCs joints, and the phase composition of the weld zone was found to be very similar to that of the parent metals. The XRD pattern advocates that the joints were fabricated with no intermetallic phases or other defects. The outcomes of this research study establish a groundwork for subsequent investigations into the mechanical and tribological behaviour of aluminium-based MMCs that have been welded using the TIG welding process.

### SEM microstructure analysis in weld zone of Al/TiB₂ MMC and Al/SiCp MMCS

**Fig. 5:**
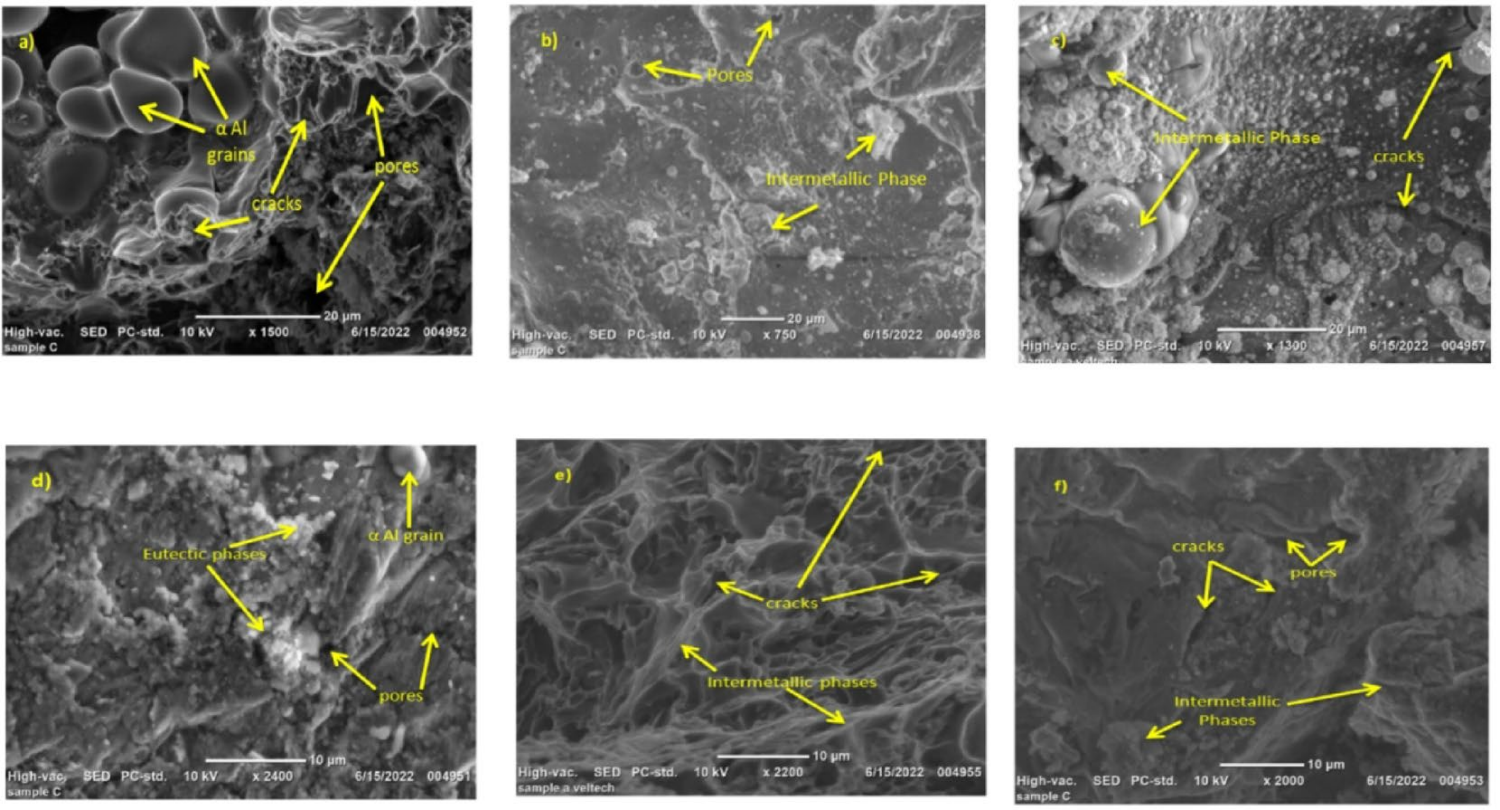
Microstructure analysis of welded MMCs with various ceramic concentration **a** 2% of SiCp; **b** 4% of SiCp; **c** 6% of SiCp; **d** 2% of TiB₂; **e** 4% of TiB₂; **f** 6% of TiB₂.

The microstructure of Al/SiCp welded joints containing 2% SiCp shows (Fig. 5a) the primary dendrites of α-Al grains and that SiCp particles are uniformly dispersed throughout the matrix, and their size ranges from 5 to 10 μm. The inclusion of Si particles in the MMCs led to the development of an intermetallic at the joint interface between the reinforcements (SiCp) and the matrix. The phase acts as a wetting agent, reducing the surface tension between the reinforcements (SiCp) and the aluminium matrix and facilitating their dispersion and bonding. The Al_3_K phase also contributes to the refinement of the microstructure by nucleating new α-Al grains. The TIG welding process introduces some changes in the microstructure by inducing local melting and solidification. Cracks and pores are observed in some areas, especially near the surface and the weld line. This may be due to the thermal stresses induced by the welding process and the variances in the coefficients of thermal expansion between the composite and the welding filler metal^29,30^. The surface confirmations the existence of Ti, O, and Mg in the weld region, which is consistent with the filler metal composition. Figure 5b displays the SEM microstructure of the welded Al/SiCp composite with 4% SiCp reinforcements. The microstructure is similar to that of the 2% composite, with the addition of more Si-containing intermetallic phases at the particle-matrix interface. The size of the SiCp particles is also slightly larger, ranging from 5 to 15 μm. XRD examination on the composite material of Al/SiCp shows sharp peaks within the Al and SiC phases. The welding procedure minimally alters the microstructure, but the defects are minimal compared to the case on the 2% composite. The detected peak on the Al and the SiC assures us that the reinforcement is preserved following the TIG welding process, and the filler metal composition is constant with the weld zone. Figure 5c shows the SEM micrograph of the TIG-welded joint of the Al/SiCp composite with 6% SiCp reinforcements. The microstructure is similar to that of the 4% TiB₂ composite, with the addition of more Si-containing intermetallic phases at the particle-matrix interface. The size of the SiCp particles is also a little larger, ranging from 10 to 20 μm that attributed to lower the TS and hardness. The TIG welding process also causes more changes in the SEM microstructure such as cracks appearing in some places, especially near the center of the specimen. The XRD phase compounds confirms the Ti, O, and Mg elements in the weld region, which matches the filler metal composition.

The SEM micrograph of the TIG welded 2% Al/TiB₂ composite is shown in Fig. 5d. The microstructure has primary dendrites of α-Al grains and the eutectic phase made up of α-Al, TiB₂ particles, and an inter metallic phase that contains Ti- and K (Al_3_Ti). The TiB₂ particles are uniformly distributed throughout the matrix, and their size ranges from 5 to 10 μm. The incorporation of TiB₂ in the composite leads to the development of Al_3_Ti at the joint interface between the aluminium matrix and the TiB₂ particles. The Al_3_Ti phase acts as a wetting agent, reducing the surface tension between the TiB₂ particles and the matrix and facilitating their dispersion and bonding. The Al_3_Ti phase also plays a crucial role to the refinement of the microstructure by nucleating new α-Al grains. The TIG welding parameters causes some changes in the microstructure by inducing local melting and solidification. Pores are observed in some areas, especially near the surface and the weld line. Thermal stress and differences in thermal coefficient are the reason for this occurrence which was already reported by^31^. The XRD phase compound confirms the presence of Mg, Si, and Fe in the weld region, which is consistent with the filler metal composition. Figure 5e shows the SEM micrograph of the TIG welded 4% Al/TiB₂ composite. The microstructure is similar to that of the 2% Al/TiB₂ composite, with the addition of more Al_3_Ti intermetallic phases at the particle-matrix interface. The size of the TiB₂ particles is also slightly larger, ranging from 5 to 15 μm. The XRD pattern evidences the simultaneous existence of the Al and TiB₂ phases within the composite by the XRD pattern. Minor microstructural differences can be seen after welding but the structure is still uniform with fewer flaws compared to the 2% sample. The XRD analysis shows that Mg, Si, and Fe are present in the weld region, which matches the filler metal composition. Figure 5f shows the SEM image of Al/TiB₂ composite joint consisting of 6% TiB₂. The microstructure characteristic was found to be very similar to that of the 4% composite, but there are more Al_3_Ti intermetallic phases at the particle-matrix interface. The TiB₂ particles are also a bit larger; their size varied from 10 to 20 μm. XRD analysis of the welded Al/TiB₂ composite reveals sharp peaks due to the Al and TiB₂ phases showing strong metallurgical bonding. HAZ exhibits some variations in the morphology of grains but the architecture is otherwise dense and defect-free consistent with the composition of the filler composition^27^.

The results emphasize that the inclusion of SiCp and TiB₂ results in the formation of Si and Ti-containing intermetallic phases, respectively, at the particle-matrix interface, which act as wetting agents and endorse the microstructure refinement. The TIG welding causes localized melting and solidification, which alter microstructure and result in defects such as cracks and pores. The defects are easily visible in the 6% SiC and 6% TiB₂ composites because their microstructure has coarser and agglomerated particles. Overall, the microstructure analysis provides insights into how tungsten-containing MMCs with different percentages of SiCp and TiB₂ as co-reinforcement change phase and form defects in the A356 alloy matrix. The results can be used to determine the best processing conditions and improve the composites for a better and wider range of applications.

### Mechanical properties of TIG welded Al/TiB₂ and Al/SiCp composites

#### Tensile properties

Table 1 shows the tensile properties of TIG-welded joints, indicating that the addition of 2% of Al/SiCp particles improved the TS by 6.7% and yield strength by 8.9% compared to the pure A356 aluminium matrix. But the elongation went down by 53.1%, and this is suitable for places where products demand high strength without low ductile properties. Adding 4% SiCp particles to Al/SiCp composites increased the TS by 15.4% when SiCp particles of 4% were added into Al/SiCp composites. In this case, the elongation was found to be decreased by 40.8%, which is comparatively good for the product’s ductile properties. When compared to aluminium base metal, the 6% inclusion of SiCp in the Al/SiCp composites displayed the highest TS, which increased by 11.7%, and the yield strength increased by 12.9%. At this proportion, the elongation decreased by 46.9%, and it is ideal for high-strength applications with moderate ductile properties. Hence, the optimum condition is that the 4% concentration of Al/SiCp composites is suitable for manufacturing light weight panels, satellite frames in aerospace and defense applications.

**Table 1:** Tensile behaviour of TIG welded composites.

Specimen	TS (MPa)	Yield Strength (MPa)	Elongation (%)
A356 parent metal	196.4	125.1	4.9
2% Al/SiCp	209.6	136.3	2.3
4% Al/SiCp	226.7	147.2	2.9
6% Al/SiCp	219.3	141.2	2.6
2% Al/TiB₂	205.2	129.5	2.1
4% Al/TiB₂	228.5	149.2	3.0
6% Al/TiB₂	218.1	138.8	2.5

**Fig. 6:**
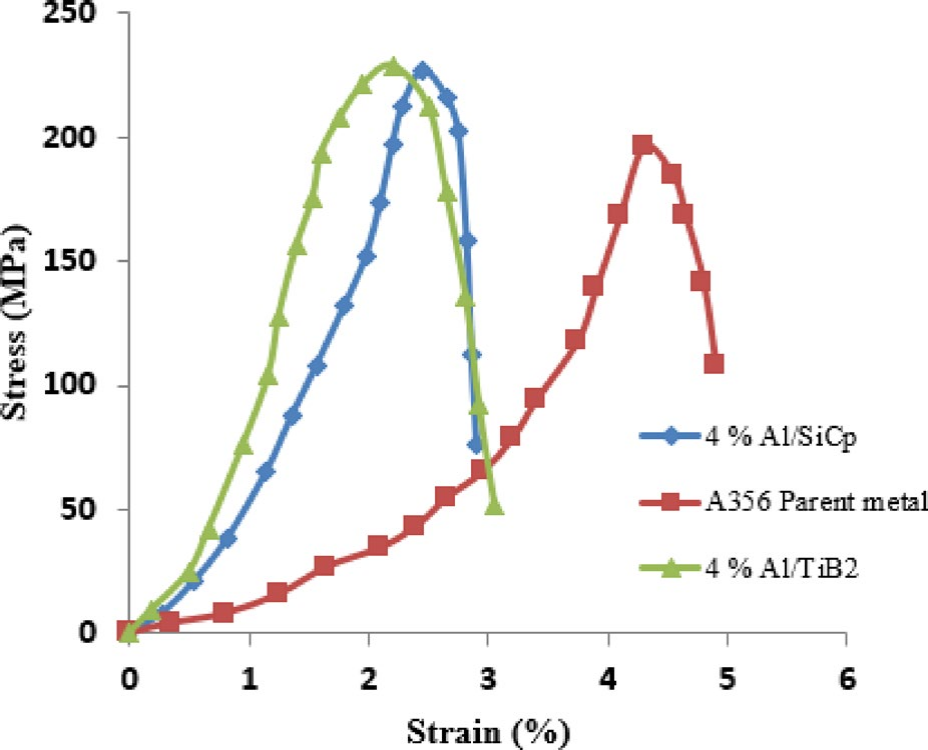
Stress–strain curve.

Adding 2% TiB₂ particles to Al/TiB₂ composites increased TS by 4.5% and yield strength by 3.5% compared to A356 base metal but reduced elongation by 57.1%. This concentration works well when moderate strength and good ductility are needed. With 4% TiB₂ particles, TS rose by 16.3% and yield strength by 19.2% while elongation dropped by 38.8%. This option suits uses that demand high strength and moderate ductility. At 6% TiB₂ particles in Al/TiB₂ composites, TS improved by 11% and yield strength by 10.9%, but elongation decreased by 49%. This composition is also suitable for applications needing high strength and moderate ductility. Overall, the combination of composites may be suitable for fabricating the structural components in aerospace, drive shafts and suspension arms in automotive, etc. The highest TS was obtained at the 4 wt% reinforcement (both case) that is directly proportional to its refined and uniformly distributed microstructure. The reduced particle density at 2 wt% offers inadequate load carrying capacity and dislocation strengthening and, therefore, does not allow a relatively high strength. On the other hand, 6 wt% reinforcement brings about particle agglomeration and porosity, which are the stress concentration and continuity reducers of the effective matrix, thus limiting the strength enhancement. The 4 wt% SiCp/ TiB₂ composites (Fig. 6). hit the optimum—enough reinforcement to support high interfacial loads transfer and grains refined without the defects caused by overcrowding—so that it gives the best tensile strength compared to all the compositions. Microstructure analysis revealed ductile dimples and cleavage facets, correlating fracture mechanisms with tensile strength variations and validating the reported mechanical property results.

The TS, yield strength and elongation values of the TIG-welded Aluminium-based MMCs joints increases as the ceramic particles’ concentration and salt flux content rise. This is because of the ceramic particles strengthen the material, while salt flux prevents the oxide film forming on the particle surfaces. Ceramic particles raise dislocation density and limit dislocation movement, which makes the composites stronger. Salt flux also helps the particles bond better with the molten aluminium matrix by stopping oxide layer formation, leading to stronger connections between the particles and the matrix^28–30^. The phases found in the MMCs joints matches the expected phase composition that normally developed when the ceramic particles added into the aluminium matrix. SiC, TiB₂ and K_2_Ti2O_5_ confirm the presence of the ceramic particles and salt flux^31^. The detection of Al_3_K_2_Si_4_O1_3_ and K6.64(Ti5.56Al0.44O17) (B_4_O_7_) suggests that complex phases formed from reactions among the ceramic particles, salt flux and matrix. Microstructure analysis showed that the ceramic particles and the matrix were evenly mixed. The ceramic particles were well distributed, which led to strong bonding obtained between the ceramics and the aluminium matrix.

**Fig. 7:**
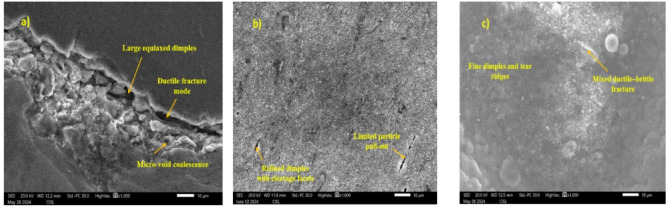
Fractographic Analysis of welded MMCs with various ceramic concentration **a** A356 parent metal, **b** 4% of SiCp; **c** 4% of TiB₂.

The fractographic examination of the tensile-fractured TIG-welded Al/SiCp and Al/TiB₂ composites was conducted by SEM in order to relate fracture mechanisms to observed tensile properties (Fig. 7a–c). The fracture surface of the A356 base metal predominantly displays large, deep equiaxed dimples, which indicate a ductile fracture mode ruled by micro-void nucleation, growth, and coalescence. This morphology accounts for the alloy’s higher elongation and comparatively lower tensile strength relative to the unreinforced material. The fracture surfaces of composites with optimal reinforcement content (4 wt% SiCp and 4 wt% TiB₂) reveal mixed-mode fracture governed by fine and shallow dimples, along with tear ridges, indicating controlled plastic deformation before the fracture event. Compared to the matrix alloy, the decrease in the dimple size clearly reflects the effective load transfer from the Al matrix to the dispersed ceramic phase. In light of the improved tensile strength recorded for these compositions, strong interfacial bonding is reflected in the presence of well-bonded SiCp and TiB₂ particles on the fracture surface, without any significant particle pull-out from the matrix. In addition to this, occasional cleavage-like facets are also noticed, indicative of localized brittle fracture caused by hard reinforcements, in agreement with the loss in ductility noted in these composites. For the higher reinforcement content of 6 wt%, fracture morphology is more brittle; this manifestation is supported by particle agglomeration, micro-cracks, cleavage facets, and signature particle debonding observed in fractographs. These provide stress concentration sites, leading to early crack nucleation and growth. These results explain the marginal reductions in tensile strength and elongation with increased reinforcement. Fractographic analysis consolidates these observations made by the tensile tests, that the highest tensile properties in the 4 wt% reinforced Al/SiCp and Al/TiB₂ composites result from refinement of microstructure, homogeneity in particle distribution, and good matrix-reinforcement interfacial bonding, while excess reinforcement promotes brittle fracture features with reduced mechanical efficiency.

#### Hardness study

**Fig. 8:**
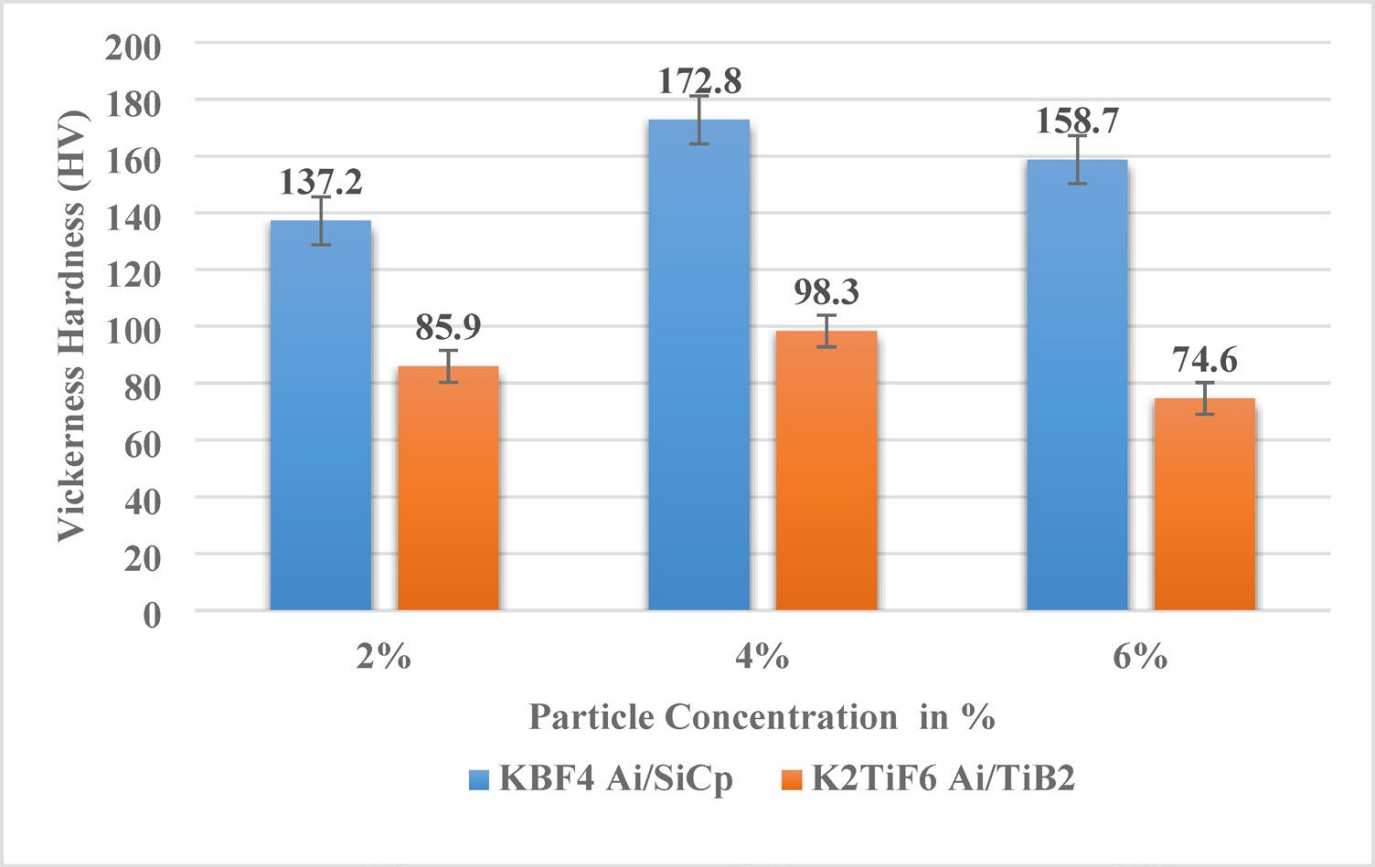
Hardness analysis.

The measured Vickers hardness value of the TIG-welded Al/SiCp composite with 2% reinforcement was 137.4 HV. The increased hardness at 2 wt% SiCp results from uniform SiC–matrix bonding and refined grain structure; TiB₂ does not form a ceramic layer around SiC (Fig. 8). This layer increases the surface area of the SiC particles, which helps them reinforce the matrix material more effectively. The larger surface area results in enhanced bonding between the SiC reinforcements and the matrix. Hence, the composite exhibited a higher hardness value due to these changes. No clustering was seen in the microstructure, which means that it shows that SiC reinforcements are evenly spread throughout the matrix, and the ceramic layer around them is visible. The measured hardness value for 4% Al/SiCp composite joint made by TIG welding was 172.8 HV. The increase of SiCp concentration leads to enhanced interfacial bonding and enables improved load transfer between the reinforcement and matrix, thereby resulting in elevated hardness levels^29,31^. The X-ray diffraction (XRD) peaks associated with the (100), (111), and (220) planes of SiC exhibit increased intensity with a higher SiCp content, thereby affirming the retention of reinforcement. The Vickers hardness of the TIG-welded composite consisting of 6% Al/SiCp was measured at 158.7 HV. At higher SiCp content (6 wt%), the composite has a denser particle distribution and better particle–matrix bonding. The microstructure is still uniform, comparable to the 2% and 4% composites but with an increased concentration of the ceramic reinforcement to yield the increased hardness. The hardness increases at 4 wt% due to optimal particle distribution and effective load transfer, whereas at 6 wt% agglomeration and porosity reduce the available load-bearing area and lower the hardness.

Vickers hardness value of the TIG-welded 2% Al/TiB₂ composite material was 85.9 HV. For this composition the reduced hardness has been due to restricted reinforcement dispersion and reduced load transfer by the aluminium matrix. The TIG-welded 4% Al/TiB₂ composite shows uniform dispersion of TiB₂ particles throughout the matrix. XRD confirms the presence of Al and TiB₂ phases without any secondary reaction products. The measured Vickers hardness of this composite was 98.3 HV, indicating effective reinforcement bonding and improved matrix strength. At an addition level of 6 wt% TiB₂, particle agglomeration is more pronounced decreasing the load-bearing area effectively available in the matrix and leading to the deterioration of the hardness^31,32^. Vickers hardness corresponding to the TIG-welded 6% Al/TiB₂ composite was measured to be 74.6 HV. The XRD patterns thus indicate that the crystalline phases of the SiC and TiB₂ reinforcement remain unaltered upon TIG welding, and no new intermetallic compound and reaction phases could be identified, validating the high chemical stability. Variability in the hardness within the six different metal matrix composites is primarily attributed to composition. Compositions with uniformly distributed hard particles within the matrix showed elevated values of hardness. Furthermore, the flux employed during the preparation also contributed to the refining of the microstructure enhancing the mechanical properties. Hardness increased gradually with elevated concentrations of the TiB₂ particles. SEM micrographs provided the evidence of even distribution of the ceramic reinforcements within the matrix, an aspect behind the composite performance enhancement. XRD results revealed the formation of intermetallic upon the addition of the ceramic reinforcements by virtue of interactions between the aluminium matrix and the reinforcement during the process. Hardness enhancement is attributed to uniform particle dispersion and minimal agglomeration observed in SEM, which promotes effective load transfer and strong interfacial bonding at 4 wt% reinforcement.

## Conclusion

This experimental study examined the influences of different reinforcement compositions on the TS, microstructure morphology, and microhardness of aluminium-based MMCs (Al/SiCp and Al/TiB₂) developed by TIG welding. The important findings can be summarized as follows:

- Ceramic reinforcements inclusion i.e. SiCp and TiB₂ were significantly affected the microstructural characteristics of the TIG-welded aluminum-based MMCs (Al/SiCp and Al/TiB₂) and improved the distribution and interfacial bonding of the reinforcements by the aluminium matrix.
- This investigation concluded that the addition of 4% SiCp and 4% TiB₂ provided the higher hardness and TS among the corresponding composites and hence established the said formulations to be optimum to obtain better mechanical properties.
- The addition of the ceramic reinforcements led to an improvement in the TS and hardness of the welds. However, the improvement entailed a slight decline in the level of ductility, highlighting the need to obtain the balance between strength and toughness for specific uses.
- From the SEM micrographs, the reinforcement particles were found to be uniform with the addition of the 2% and the 4% SiCp and TiB₂ particles, whereas minor agglomeration and porosity were noticed at 6% reinforcement in both cases.
- XRD analysis confirmed the preservation of the Al–SiC and the Al–TiB₂ phases upon TIG welding, validating steady interfacial bonding between the aluminium matrix and the ceramic particles without the generation of any undesirable secondary phases.
- Results are critical to the fine-tuning of reinforcement composition to achieve greater strength and structure integrity with sufficient ductility to be useful to the field of engineering.

## Data Availability

All data generated or analysed during this study are included in this published article.

## References

[1] Kamboj, N. & Thakur, L. A study of the processing and characterization of RSM optimized YSZ-Inconel625 wear-resistant TIG weld cladding. *Surf. Topogr. Metrol. Prop.* **10** (4), 045021 (2022).

[2] Selvamani, S. T., Bakkiyaraj, M., Palani, S. & Yoganandan, G. Corrosion behavior and analysis on friction stir welded aluminium matrix composites. *Surface Topogr. Metrol. Prop.* **10**, 025036. 10.1088/2051-672X/ac7a50 (2022).

[3] Cheng, Y. et al. Studies on microstructure and properties of TiB₂–Al3Ti ceramic particles reinforced spray-formed 7055 aluminium alloy fusion welded joints. *J. Mater. Sci. Technol.* **19** 1298 (2022).

[4] Huang, T. et al. Appropriate amount of TiB₂ particles causes the ductile fracture of the un-weldable spray-formed 7055 aluminium alloy TIG-welded joint. *Mater. Res. Express* **8** 096512 (2021).

[5] Bo, W. A. N. G., Xue, S. B., Han, Y. L. & Lin Z. Q. Effect of combinative addition of Ti and Sr on modification of AA4043 welding wire and mechanical properties of AA6082 welded by TIG welding. *Trans. Nonferrous Met. Soc. China* **27** 272 (2017).

[6] Huang, T. et al. Study on ductile fracture of unweldable spray formed 7055 aluminium alloy TIG welded joints with ceramic particles. *Mater. Today Commun.* **29** 102835 (2021).

[7] Rajaravi, C., Ganesh, B., Lakshmanan, S. & Gobalakrishnan, B. Influence of TIG welding processing parameters on mechanical properties of austenitic stainless steel using Taguchi analysis. *Mater. Today Proc.* **72** 2402 (2023).

[8] Qin, Q. D., Huang, B. W., Wu, Y. J. & Su, X. D. Microstructure and mechanical properties of friction stir welds on unmodified and P-modified Al-Mg2Si-Si alloys. *J. Mater. Sci. Technol.* **250** 320 (2017).

[9] Fattahi, M., Ghaheri, A., Arabian, N., Amirkhanlu, F. & Moayedi, H. Applying the ultrasonic vibration during TIG welding as a promising approach for the development of nanoparticle dispersion strengthened aluminiumweldments. *J. Mater. Sci. Technol.* **282** 116672 (2020).

[10] Dinaharan, I., Kalaiselvan, K., Akinlabi, E. T. & Davim, J. P. Microstructure and wear characterization of rice husk Ash reinforced copper matrix composites prepared using friction stir processing. *J. Alloys Compd.* **718** 150 (2017).

[11] Ahmadi, E. et al. Microstructure and mechanical properties of Al/ZrC/TiC hybrid nanocomposite filler metals of tungsten inert gas welding fabricated by accumulative roll bonding. *J. Manuf. Process.* **26** 173 (2017).

[12] Amirizad, M. et al. Evaluation of microstructure and mechanical properties in friction stir welded A356 + 15% SiCp cast composite. *Mater. Lett.* **60** 565 (2006).

[13] Ramkumar, K. R. & Natarajan, S. Investigations on microstructure and mechanical properties of TiO2 nanoparticles addition in al 3003 alloy joints by gas tungsten Arc welding. *Mater. Sci. Eng. A* **727** 51 (2018).

[14] Wang, X. H., Song, S. L., Qu, S. Y. & Zou, Z. D. Characterization of in situ synthesized tic particle reinforced Fe-based composite coatings produced by multi-pass overlapping GTAW melting process. *Surf. Coat. Technol.*, **201** 5899 (2007).

[15] Fattahi, M., Nabhani, N., Rashidkhani, E., Fattahi, Y., Akhavan, S. & Arabian, N. A new technique for the strengthening of aluminium tungsten inert gas weld metals: using carbon nanotube/aluminium composite as a filler metal. *Micron* **54** 28 (2013) 10.1016/j.micron.2013.07.004 23948441

[16] Varshney, D. & Kumar, K. Application and use of different aluminium alloys with respect to workability, strength and welding parameter optimization. *Ain Shams Eng. J.* **12** 1143 (2021).

[17] Reddy, M. I., Madhavarao, S., BhadriRaju C, R., Kumar, M. A. & Varma, P. R. The effect of carbon fiber powder reinforced composite coating on mechanical properties of TIG welded black steel pipes. *Mater. Today Proc.* **62** 3516 (2022).

[18] Chen, Y. B., Miao Y. G., Li L. Q. & Lin, W. U. Joint performance of laser-TIG double-side welded 5A06 aluminium alloy. *Trans. Nonferrous Met. Soc. China* **19** 26 (2009).

[19] Bo, W., Xue, S. B., Han, Y. L. & Lin, Z. Q. Effect of combinative addition of Ti and Sr on modification of AA4043 welding wire and mechanical properties of AA6082 welded by TIG welding. *Trans. Nonferrous Met. Soc. China* **27** 272 (2017).

[20] Liu, Z. et al. Effect of filler wire on mechanical properties, microstructure and natural aging behavior of 2A55 Al-Li alloy TIG welded joint. *Metals* **13** 347 (2023).

[21] Dhilip, A. & Nampoothiri, J. Investigating the effects of ultrasonic assistance on TIG welding of AA7075 alloys: a machine learning-based optimization study using RSM-PSO. *Phys. Scr.* **100** (1), 016002 (2024).

[22] Gupta, N. K., Pyla, K. R., Debta, M., K & Masanta, M. Performance evaluation of TIG cladded in-situ TiC-TiB₂ composite coating fabricated on AISI304 stainless steel. *Mater. Today Proc.* **62** 5956 (2022).

[23] Buytoz, S. & Ulutan, M. In Situ synthesis of SiC reinforced MMC surface on AISI 304 stainless steel by TIG surface alloying. *Surf. Coat. Technol.* **200** 36984. (2006).

[24] Gurijala, C., Rajendran, R. & Giridharan, K. Investigation on effects of nano-reinforcement on the mechanical properties, fatigue, and microstructural analysis of dissimilar AA6061-Mg AZ31B weld joints. *Phys. Scr.* **99** (11), 115928 (2024).

[25] Nanjundan, A., Natarajan, U. & Simson, D. Investigation of microstructure and mechanical properties of Al-Si alloy thin-walled cylindrical part fabricated by CMT based WAAM process. *Phys. Scr.* **99** (10), 105606 (2024).

[26] Gao, Z. et al. Experimental study on the low-power laser-TIG hybrid welding of SiCp/6061-T6 al matrix composites. *Opt. Lasers Eng.* **186**, 108861 (2025).

[27] Shi, Y., Wang, S. & Jian, Y. Inhibition mechanism of the interface reaction in the molten pool of SiCp/Al composites by pulsed laser welding with powder-filling. *Chin. J. Mech. Eng.* **37** (1), 154 (2024).

[28] Senthilkumar, J., Bakkiyaraj, M., Balasubramanian, M. & Loganathan, T. G. Effect of FW conditions on mechanical and microstructural characteristic of AA6061/SiC/Graphite hybrid composites joint by empirical relationship. *Surf. Topogr. Metrol. Prop.* **9** (4), 045042 (2021).

[29] Wang, Z. et al. Effects of laser welding parameters on the porosity and acicular phase in SiCp/6092 aluminum matrix composite welded joints. *J. Mater. Res. Technol.* **23**, 5127–5141 (2023).

[30] Biswas, K. & Sahoo, C. K. A review on TIG cladding of engineering material for improving their surface property. *Surf. Topogr. Metrol. Prop.* **11** (2), 023001 (2023).

[31] Chittoriya, B. S., Jayant, A. & Kumar, R. Effect of multipass FSP and (SiC + TiB₂) nanoparticles on the mechanical and metallurgical characteristic of the hybrid metal matrix composite. *Silicon* **15** (18), 7927–7941 (2023).

[32] Lin, F. et al. Microstructure, mechanical and thermal-properties of ultrafine-grained Al2024–TiC-GNPs nanocomposite. *Mater. Sci. Eng. A* **841**, 142855 (2022).

